# CareACT - internet-based intervention for enhancing the psychological well-being of elderly caregivers – a study protocol of a controlled trial

**DOI:** 10.1186/s12877-019-1071-9

**Published:** 2019-03-05

**Authors:** Päivi Lappalainen, Inka Pakkala, Riku Nikander

**Affiliations:** 1 The GeroCenter Foundation for Aging Research and Development, Jyväskylä, Finland; 2 Department of Psychology, University of Jyväskylä, Jyväskylä, Finland; 3 Department of Research & Education, Central Hospital of Central Finland, Jyväskylä, Finland; 4 Faculty of Sport Sciences, University of Jyväskylä, Jyväskylä, Finland

**Keywords:** Family caregivers, Depressive symptoms, Psychological well-being, Internet-based intervention, Acceptance and commitment therapy

## Abstract

**Background:**

The rapid increase in the number of elderly family caregivers underlines the need for new support systems. Internet-delivered psychological interventions are a potential approach, as they are easy to access for family caregivers who are often homebound with their care recipient. This study examines the relative effectiveness of an internet-based acceptance and commitment therapy (ACT) intervention or a standardized institutional rehabilitation program, first, in reducing depressive symptoms, and second, in improving the well-being and quality of life of elderly family caregivers compared to a control group receiving support from voluntary family caregiver associations.

**Methods:**

156 family caregivers aged 60 or more are studied in a quasi-experimental study design that compares three groups of family caregivers (Group 1; *n* = 65: a guided 12-week web-based intervention; Group 2, *n* = 52: a standardized institutional rehabilitation program in a rehabilitation center; Group 3, *n* = 39: support provided by voluntary caregiver associations). Data collection is performed at three time-points: pre-measurement and at 4 months and 10 months thereafter. Caregivers’ depressive symptoms as a primary outcome, and perceived burden, anxiety, quality of life, sense of coherence, psychological flexibility, thought suppression, and personality as secondary outcomes are measured using validated self-report questionnaires. Physical performance and user experiences are also investigated. Between-group differences in the effects of the interventions are examined using multiple-group modeling techniques, and effect-size calculations.

**Discussion:**

The study will compare the effectiveness of a novel web-based program in reducing depressive symptoms and improving the psychological well-being of elderly family caregivers, or a standardized institutional rehabilitation program representing usual care and a control group receiving support offered by voluntary caregiver associations. The results will expand the knowledge base of clinicians and provide evidence on effective strategies to improve the mental health and overall quality of life of elderly family caregivers.

**Trial registration:**

The study was retrospectively registered in www.clinicaltrials.gov (ClinicalTrials.gov Identifier: NCT03391596 on January 4, 2018.

## Background

An aging population requires the provision of both formal and informal long-term care [1, 2]. Formal care refers to health and social care provided by professionals and informal care to care given by a family member on a voluntary basis [2]. Informal care is especially of important from the economic standpoint and hence sustainability of the health care system [3, 4]. Family caregivers play an essential role in providing spouses and close ones, among others, with daily care and assistance [4]. However, providing care can have a negative effect on the caregivers’ physical and psychological health. Spousal caregivers are particularly vulnerable as they often live with their care recipient, and often face chronic health challenges of their own. Many have depressive symptoms [5]. Caregiving may also be highly rewarding emotionally [6]. Nevertheless, despite its positive aspects, the negative impact on caregivers and how this might be ameliorated merit further investigation [5].

Most of the previous traditional interventions on family caregivers’ psychological, physical and social wellbeing have addressed the caregivers of care recipients with dementia [7]. Individual and family counseling, psychoeducational programs, skills training programs and multi-component intervention programs have shown potential in improving caregiver mood, quality of life and delaying institutionalization of the care recipient [7–9]. Technology-driven programs, delivered via, e.g. phones, smart phone applications, videoconferencing and the internet are a rapidly expanding means of supporting family caregivers at lower costs and with improved accessibility [5, 10, 11]. Preliminary data suggest that internet-based interventions can reduce depression and caregiver burden and enhance quality of life similar to the same extent as traditionally delivered interventions [5, 10–12].

Acceptance and Commitment Therapy (ACT) is a relatively new form of cognitive behavioral therapy [13]. Its core message is: accept what is out of your personal control, and commit to actions that improve and enrich your well-being and life. ACT focuses on increasing psychological flexibility through acceptance, mindfulness and value processes [14]. Several meta-analyses support its effectiveness across a range of psychological problems [15–17]. A randomized trial that investigated ACT versus cognitive-behavioral therapy (CBT) for family caregivers of patients with dementia found ACT to be as beneficial as CBT [18]. Further studies are required to confirm the effectiveness of ACT-based interventions among elderly caregivers. To the best of our knowledge, no trials thus far have investigated the effectiveness of web-based ACT interventions in enhancing the well-being of elderly family caregivers.

## Aim and main hypotheses

The study aim is to investigate the relative effectiveness of 1) a guided Acceptance and Commitment Therapy (ACT) -based online psychological intervention and 2) a standard rehabilitation provided by rehabilitation centers in, first, reducing depressive symptoms, and second, in improving well-being and quality of life of elderly family caregivers, compared to 3) an active control group receiving support from voluntary family caregiver organizations. The study is thus a quasi-experimental trial comparing three groups of caregivers (Group 1; *n* = 65: guided 12-week web-based intervention; Group 2, *n* = 52: standardized rehabilitation program in a rehabilitation center; Group 3, *n* = 39: support given by voluntary family caregiver organizations). The same measurements will be conducted in all three groups, and between group differences in changes, primarily in depressive symptoms, but also, in experienced burden, anxiety, quality of life, sense of coherence, psychological flexibility, thought suppression, and personality will be studied using self-report questionnaires at pre-measurement, and at 4 and 10 months thereafter. Physical performance at pre-measurement will also be investigated. In our controlled trial, we hypothesize that the guided web-based ACT intervention will be equally effective in alleviating depressive symptoms and perceived burden and in improving mental well-being and quality of life as the standardized rehabilitation program but superior to support provided by voluntary family caregiver organizations. In addition to the aforementioned controlled trial, we aim to identify demographics and psychological variables (age, gender, personality, depressive mood, experienced burden, quality of life, sense of coherence, psychological flexibility, and suppression of thoughts) that could predict change over time (at pre-measurement, at four and 10 months thereafter). A further aim is to examine potential mediators, including psychological flexibility and suppression of thoughts, on the effects of the interventions Moreover, we aim to study user experiences and satisfaction with the web-based program, that is, how family caregivers experience and accept the web-based ACT intervention.

## Methods/design

### Study design

**Fig. 1:**
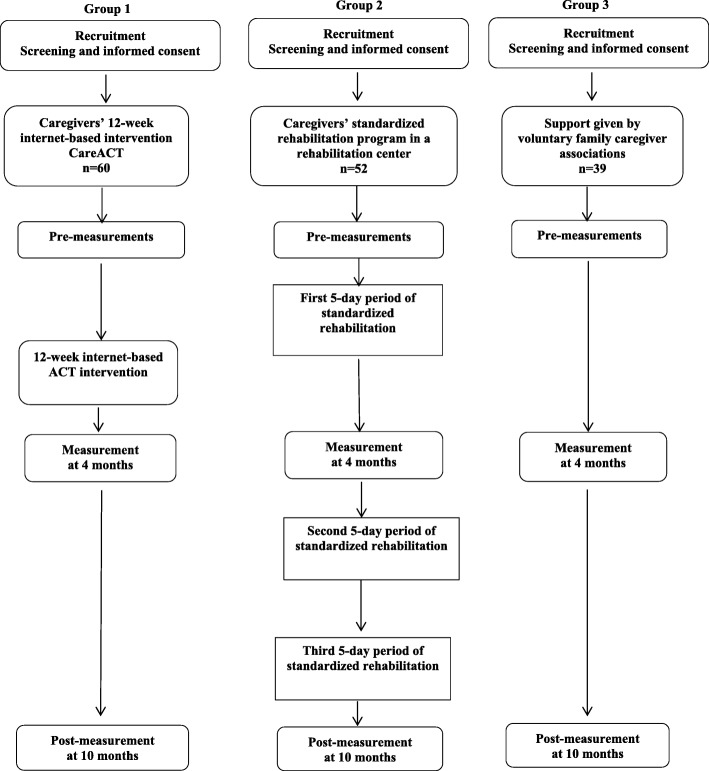
Flow chart of the study design

This study is a quasi-experimental controlled trial comparing three groups of caregivers. Group 1 is an experimental group receiving an ACT-based, guided internet intervention. Group 2 is an active comparator group receiving a standardized rehabilitation program in the rehabilitation center (usual care). Group 3 is a control group receiving support given by voluntary family caregiver associations. Data will be collected at three time points; pre-measurement, and at four and 10 months thereafter. Outcome measures are depressive symptoms (the primary outcome), caregivers’ burden, anxiety, quality of life, sense of coherence, personality, psychological flexibility, suppression of thoughts, and physical performance. We are also interested in user experiences of the novel web-based intervention. The study design with intervention arms is shown in detail in the flow chart presented in Fig. 1.

The protocol follows the Standard Protocol Items: Recommendations for Interventional Trials (SPIRIT) 2013 statement.

Data are collected from three Finnish rehabilitation centers and five voluntary family caregiver associations. The study has been approved by the ethics committee of the Central Finland Health Care District (Approval Number 3E/2016), and registered with ClinicalTrials.gov with the identifier NCT03391596. The study and data is conducted in phases to improve the flow of the trial. The first phase of the web-based ACT intervention group (Group 1) started in January 2017 in the city of Jyväskylä, and the second phase in September 2017 in the city of Tampere. The study includes pre-measurements before the interventions, measurements 4 months after the pre-measurements, and post-measurements 10 months after the pre-measurements. Pre-measurements for all groups were carried out between January and December 2017. The last post-measurements will be collected in October 2018.

### Study population

A total of 156 family caregivers (Fig. 1) aged 60 years or over reporting caregiving burden and/or depressive symptoms were recruited.

### Procedure for recruitment

The experimental group (Group 1) was recruited using advertisements in local newspapers. Interested participants were requested to call or email the members of the research team at the GeroCenter Foundation. Potential participants were informed by phone about the study and, those willing to participate, asked to give their verbal informed consent after which they were screened to ensure they met the basic inclusion criteria. After this initial screening, a structured interview was performed to check the eligibility criteria in detail.

Inclusion criteria for the experimental group (Group 1) were 1) at least 60 years of age, 2) perceived depressive symptoms and/or psychological distress, and 3) the possibility to use a computer with internet connection or willingness to use a tablet provided by the study. The exclusion criteria included 1) diagnosed severe mental disorder and 2) parallel psychological treatment. Screening for distress and/or depressive symptoms was implemented by telephone using four questions based on the DEPS screening instrument [19]. The questions were: During the last 4 weeks, how much have you been bothered by any of the following problems: 1) Have you suffered from sleeping difficulties? 2) Have you been bothered by feeling down, depressed? 3) Have you been bothered by feeling tired, or having little energy? and 4) Have you been bothered by thoughts that all the joy has gone from your life? The caregiver was included in the study if scored at least 1 point on the 4-point response scale for each question. The response scale was as follows: Not at all (0 points), To some extent (1 point), Quite a lot (2 points), Very much (3 points). Participants who passed the screening were assigned to the intervention group (Group 1).

Participants in Group 2 and 3 were recruited by contacting rehabilitation centers and voluntary family caregiver associations by email. We asked if it would be possible for members of the research group to meet potential participants at these centers/associations to inform them about the study. To be eligible for participation, family caregivers had to meet the inclusion criteria, but none of the exclusion criteria. The inclusion criteria were 1) at least 60 years of age, 2) symptoms of psychological distress and burden as determined by a physician’s examination (Group 2) and self-report (Group 3). The exclusion criteria were the same as for Group 1. Participation in the study was voluntary. A written and signed informed consent was obtained from all participants before the start of the pre-measurements.

### Interventions

#### The guided ACT-based internet intervention

**Fig. 2:**
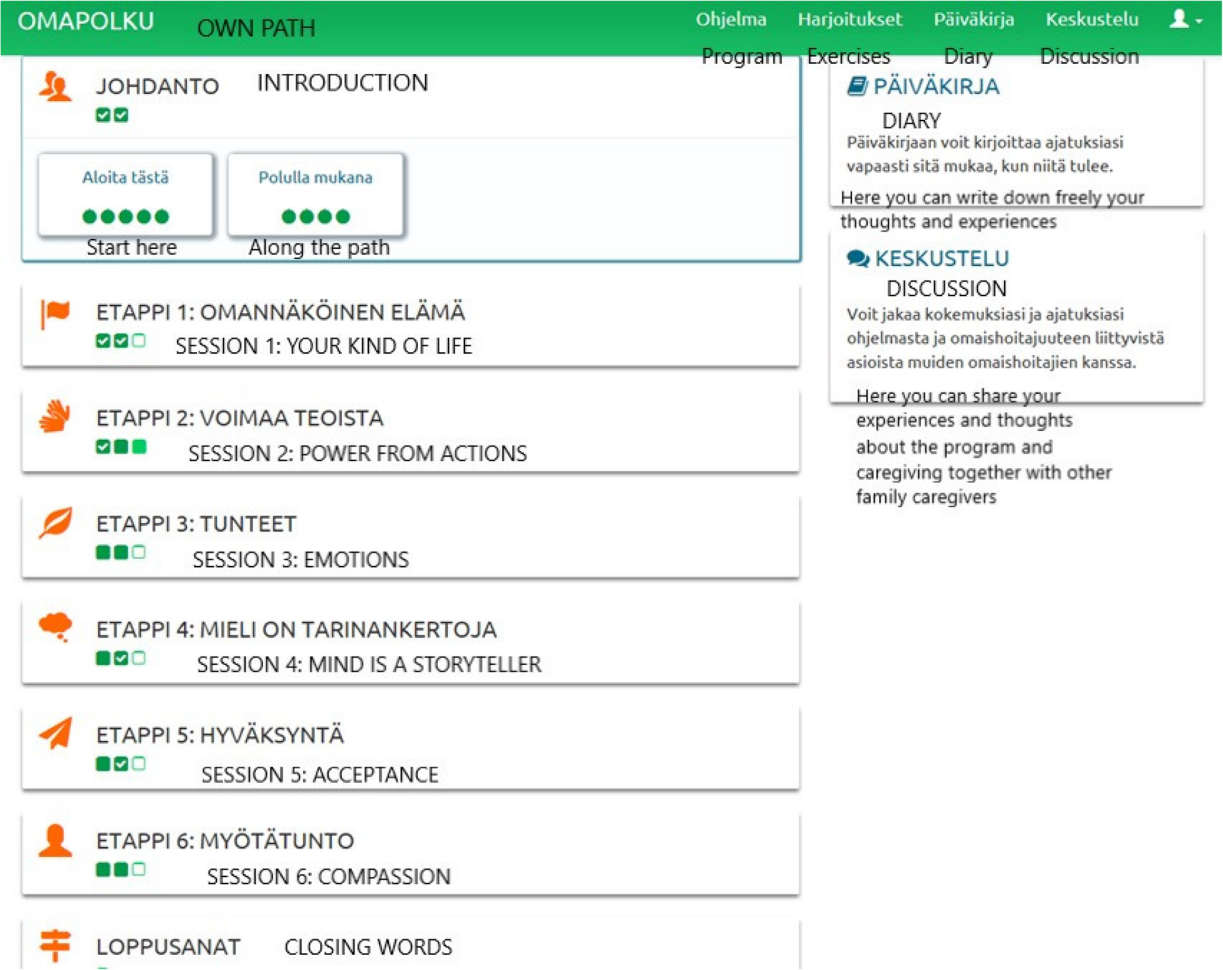
Screenshot of the internet program: Overview of the program and the six modules

**Fig. 3:**
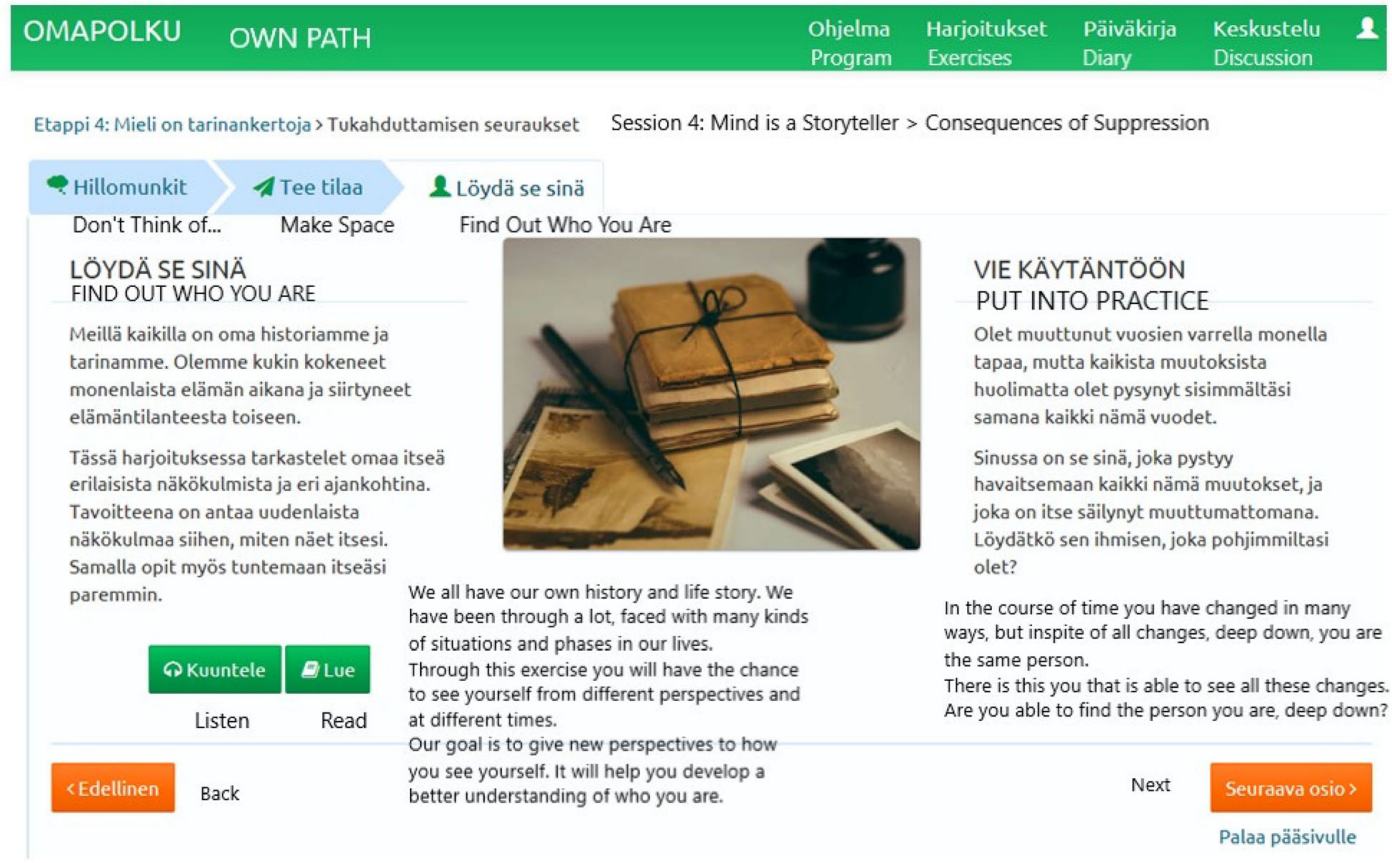
Screenshot of the internet program: Page of Step 4 with a listening exercise

The guided internet-based intervention was a 12-week program aimed at decreasing caregivers’ depressive symptoms and enhancing their psychological well-being (see Figs. 2 and 3 for screen shots of the program).

In addition to the introduction and closing module, the program content was divided into six progressive modules dealing with the core processes of acceptance and commitment therapy [13, 14]: 1) *Values*, which are chosen areas of life (family, career, friends etc.) that we personally consider important and meaningful for us and that motivate us. By pursuing our values and goals derived from our values we can live a meaningful and vital life; 2) *Value-based actions,* which are actions guided by your personal values. Committed action means taking concrete action guided by your values - doing what matters - even this is difficult or uncomfortable. Value-based actions involve steps based on short and long-term behavior change goals; 3) *Present moment* meaning being able to live in the present moment and not so much in “head”, but instead engaging in what you are doing rather than getting stuck in your thoughts and feelings. It is about practicing the skill of noticing distressing thoughts and feelings, and meeting them with acceptance; 4) *Self as Context,* which refers to “mind”, the part of you that is responsible for awareness and attention, the observing self - the part of your mind that is aware of whatever you are thinking or feeling or doing at any moment. 5) *Defusion* which means learning to step back or detach from unhelpful thoughts and worries and memories: instead of getting caught up in your thoughts, you learn to step back and watch your thinking, so that you can respond effectively - instead of getting lost inside your thoughts; 6) *Acceptance* which means making space for distress, painful feelings and sensations. You learn how to stop struggling with them, give them some space, and allow them to be present without getting overwhelmed by them. A module including compassion and self-compassion was also added to the program. The internet program contained text, 26 experiential exercises in text and audio format, a video clip for each module, a diary and a discussion forum (Table 1).

**Table 1:** Content of the ACT-based internet intervention

Topic of the module	Content	Home assignment (every 2 weeks)
Introduction	Introduction to the program Video: A caregiver telling about his role as a caregiver Exercise*: Find joy in everyday life*	
Step 1. Your Life (Values)	Text: What is important in caregiving? How can I take care of myself? Video: *What are values?* Exercises: *Curtains in the window* *The most important things in life* *Value domains*	What makes your life worth living? How do you need to take care of yourself? Do you do things that bring you strength and joy? If some of your dreams were to come true, what would they be?
Step 2. From Words to Actions (value-based actions and barriers)	Text: Even small steps count, loneliness, cherish your friendships Exercises: *Do it now!* *Obstacles on your path*	Listen to the exercise *Take action* and reflect on what small steps you could take now to improve your own wellbeing and the wellbeing of other people close to you. What could you do today, tomorrow or next month? Apply the *Obstacles*-exercise when you notice that your thoughts prevent you from doing something. What do you observe? If you wish, you can also reflect on what loneliness means to you? What do you do to alleviate it?
Step 3. Feelings: Learn to notice your feelings and be more accepting towards them (present moment)	Text: Feelings in caregiving: learn to be more aware of your feelings. Learn to notice them and to cope with them. Video: *Present moment* Exercises: *Follow your breathing* *Mindful listening* *Mindful eating* *Mindful walking* *Mindful sitting*	Choose a chore or task every day and do it this time mindfully, with more awareness and concentration. Choose something that brings you joy or something you don’t like, such as washing the dishes, cleaning, listening to music, drinking coffee or tea, eating, going for a walk etc. What small steps can you take during the next two weeks? Continue with noticing your thoughts and feelings. Have the following questions on hand and ask them of yourself when you notice unwanted private experiences: Feelings: What do I feel right now? Thoughts: What thoughts are related to these feelings? Body sensations: What sensations do I feel in my body? Situations: In what situations do I have these feelings? Actions: What do I do when I have these feelings? Do I try to change it or suppress it? Acceptance: Can I just let those feelings be without trying to push them away? I can experience all kinds of feelings and it is normal.
Step 4. Mind is a storyteller (Defusion and Self-as-Context)	Text: Observing your thoughts without being caught up in them. Video: Watch your thinking Exercises: *Weather and sky* *Observer* *Don’t think of …* *Make room for your thoughts and emotions* *Find Yourself*	When you notice that you are worrying try some of the following: Treat your mind as a separate person. Thank your mind when you notice it worrying: “Thank you for your contribution.” “What have you come up with now?” “Well, what an interesting thought!” Take an observer stance by trying out the *Observer*-exercise. What is your value-based action during this step? Describe what you did and what the experience was like. If you have time, choose a couple of the exercises provided and try them out. Notice how you can now see your thoughts in a new light and realize that you don’t have to obey them.
Step 5. Acceptance	Text: Learn to live with your unwanted guest: the illness of your nearest, the situation where you are in right now. Accept what you cannot change, accept your feelings and thoughts Video: What is acceptance? Exercises: *The broken machine* *Observer (short version)* *Bird nest*	Reflect on whether there is anything in your life which you are currently struggling with. Is there something related to caregiving that you would need to practice so that you would open up and accept it? Choose one such thing and practice it. See what happens and describe it. What step could you take this time? Do it and describe your experiences.
Step 6. Compassion	Compassion towards yourself (self-compassion) and towards others, gratitude for small things in life	Keep a gratitude diary for a week. Do both or one of the following: Compassion: Try to remind yourself of things that made you feel down or moments when you criticized yourself. Write them down. Take them one by one and practice taking an accepting and friendly stance towards them. Feel compassion towards yourself. Gratitude: Write down three things that you are thankful for during the day or in your life in general. It can be a tiny thing, a good day with your care-recipient, a nice, relaxing moment having coffee, a beautiful song, sunshine or a nice word from someone. Try to find something every day.
Closing words	The journey continues Online questionnaire (well-being) The next steps	

A pre-measurement package including information on basic demographics, five self-report questionnaires, a detailed information handout on the study procedures including ethical principles, and a written informed consent in two samples were sent to prospective participants of the guided web-based intervention (Group 1) via regular mail. They were asked to familiarize themselves with the information sheet*,* and, if they were willing to participate*,* to sign the informed consent and fill in the questionnaires provided with the letter. Participants were also encouraged to telephone the research team should they have any queries related to the study. A total of 65 family caregivers were recruited to the guided ACT-based internet intervention.

Each participant in the web-based group was offered a support person who subsequently contacted the participant via telephone and scheduled an initial 2-h interview meeting. The interview was arranged according to each participant’s preferences either at their home, or University of Jyväskylä premises. The interview consisted of a brief overview of the study and possible questions related to the study were answered. Participants’ willingness was confirmed and the signed informed consent collected. Participants were then asked to fill in the rest of the psychological measurements. The support person administered the Short Physical Performance Battery (SPPB) [20], which included gait speed, chair stand and balance tests, to assess the participant’s physical function. A structured interview (45–50 min) including 30 questions related to the participant’s current situation was conducted. The interview questions were adapted from the psychosocial interview template [21, 22]. By the end of the initial meeting, the support person introduced and gave an overview of the web-based program and ensured that the participant had access to the program. Participants used either their own PC or a tablet provided by the study. Participants were also given a short instruction sheet that contained the intervention timetable and instructions on how to use the program.

The personal support person communicated with the participant by telephone every other week (a total of six telephone calls). Participants were advised to spend two weeks on each prescribed module for 12 weeks. Thus, the telephone calls were scheduled for the end of each module. The telephone calls were restricted to a maximum of 20 min per two weeks and guided by semi-structured phone call scripts. The purpose of the phone calls was to provide feedback based on entries made by the participant, check the participant’s situation, to provide an opportunity for the participant to discuss any issues arising from the home assignments, and to encourage the participant to continue working on the assignments.

The four-month measurement was scheduled at 14 weeks from the pre-measurement. Via regular mail, participants received a measurement package via regular mail including five psychological questionnaires, a questionnaire about the acceptability of the web-based program and an additional form containing nine questions about the intervention. Subsequently, the support person telephoned the participant and scheduled a meeting at either the participant’s home or the University. The meeting followed the same structure as the initial meeting and included the remaining questionnaires, the Short Physical Performance Battery (SPPB) measurement, and a semi-structured interview with nine questions. After the 12-week web intervention had finished, participants continued to have access to the program until the 10-month post-measurement. The post-measurement will follow the same procedure as the pre- and 4-month measurements.

#### Support persons

Psychology and health science students from the University of Jyväskylä act as support persons during the web-based ACT intervention. All of them receive a short training in ACT approach, totally eight hours, and familiarize themselves with the ACT literature. The student support persons also participate in three supervision sessions (3 × 2 h) during the 12-week intervention.

#### Standardized rehabilitation - Care provided by rehabilitation centers

A standardized institutional rehabilitation program provided by certificated rehabilitation centers was selected for comparison with the guided ACT-based intervention. In this study, the institutional rehabilitation program represents the usual care available to family caregivers in Finland. These rehabilitation courses for family caregivers are funded by the Social Insurance Institution (SII) and intended for people who work daily as family caregivers [23] and who experience symptoms of exhaustion or whose ability to function may be compromised due to their own illness or perceived caregiving burden [23].

Two types of rehabilitation courses for family caregivers are available. Individual rehabilitation courses which are intended for family caregivers only last 10 days (5 + 5 days) altogether and are carried out across 6–8 months. Courses intended for both caregiver and care recipient last for a total of 15 days and are organized in three 5-day periods (5 + 5 + 5 days) over a 10-month period [23]. Family caregivers can apply for a rehabilitation course by filling in the appropriate form and enclosing a physician’s statement on the need for rehabilitation with the application form. The physician’s statement indicates the caregiver’s the illness or impairment, describes the applicant’s functional status, and makes a recommendation, along with the reasons, for the type of rehabilitation to be implemented. One condition for rehabilitation is that it should have the potential to improve or maintain the caregiver’s ability to function [23]. Rehabilitation courses are free of charge for the participants.

The rehabilitation courses involve a multidisciplinary team whose composition varies depending on the course format. In general, the team is composed of a physician, a nurse, a physiotherapist, a psychologist, an occupational therapist, and a social worker. Most of the activities during the rehabilitation program are carried out in groups. The methods used include discussion and counseling, assignments to be completed between the rehabilitation sessions, a rehabilitation diary, operational methods and information about caregiver counseling and support services [23].

The study team contacted rehabilitation centers arranging rehabilitation courses for caregivers and asked if they were willing to cooperate in the study. Three rehabilitation centers replied in the affirmative and gave their consent. Caregivers were recruited from rehabilitation courses held between April and October 2017. At the beginning of each rehabilitation course, the rehabilitation center informed the caregivers about the study. Interested caregivers attended a meeting where the research team was present and gave a more detailed description of the study. Caregivers’ questions about the study were answered and those willing to participate were asked to fill in the consent form in two samples. The Short Physical Performance Battery (SPPB) was administered, and caregivers who returned the consent form received a measurement package and were asked to return the measurement package in regular post. A total of 52 family caregivers were recruited from the standardized rehabilitation program.

#### Support provided by voluntary family caregiver associations

Caregivers from voluntary family caregiver associations, mostly giving peer support, formed a control group. Family caregiver associations support family caregivers by providing individual counseling, information about services, tools, and resources, such as peer support groups, events, trips and open cafés. The family caregiver controls were recruited from five voluntary caregiver associations. The research team contacted the associations via email, and, after obtaining permission, attended the family caregiver support group or other activities of the association to inform members about the study, and to recruit participants. The family caregivers who gave their informed consent to participate in the study, filled in the measurement package. In addition, a short physical performance test (SPPB) was performed at pre-measurement. Recruitment was implemented from March to December 2017. The mid-measurement package was sent to the caregivers at four months and the post-measurements will be carried out at 10 months after the pre-measurements. A total of 39 family caregivers were recruited from voluntary caregiver associations.

### Measurements

Differences in changes between the groups will be examined at four months and at 10 months (post-measurement) after the beginning of the study (pre-measurement). All the participants are measured at the beginning of the study (pre-measurement), at 4 months, and at 10 months (post-measurement) (see Table 2).

**Table 2:** Measures included in the study

Measure	Subscales	Pre	4-months	10-months	Ref.
Main outcome measure					
Depression Beck Depression Inventory (BDI-II)		x	x	x	[24]
Secondary outcomes					
Perceptions of the caregiver role Carers of Older People in Europe (COPE Index)	Negative impact	x	x	x	[26]
	Positive value of caregiving				
	Quality of support				
Anxiety Generalized Anxiety Disorder (GAD-7)		x	x	x	[28]
Quality of Life: WHO Quality of Life-BREF (WHOQOL-BREF)	Physical health	x	x	x	[29]
	Psychological health				
	Social relationships				
	Environment				
Sense of coherence Sense of coherence (SOC-13)	Comprehensibility	x	x	x	[31, 32]
	Manageability				
	Meaningfulness				
Psychological Flexibility Acceptance and Action Questionnaire (AAQ-II)		x	x	x	[33]
Experiential Avoidance The Experiential Avoidance in Caregiving Questionnaire (EACQ)	Active Avoidant Behaviors	x	x	x	[35]
	Intolerance of Negative Thoughts and Emotions Towards the Relative				
	Apprehension Concerning Negative Internal Experiences Related to Caregiving				
Thought suppression The White Bear Suppression Inventory (WBSI)		x	x	x	[36]
Personality The ‘Short Five’ (S5)	Neuroticism	x	x	x	[38]
	Openness				
	Conscientiousness				
	Extroversion				
	Agreeableness				
Physical performance Short Physical Performance Battery (SPPB)	Balance, 4 m walking, chair rising	x	x ^Group 1 only^	x ^Group 1 only^	[20]
User experiences				x ^Group 1 only^	

### Outcome measures

#### Main outcome measure

##### Depression

The Beck Depression Inventory [24] will be used as the main outcome measure. It is commonly used in research and clinical practice to measure the presence and severity of depression. It contains 21 questions on depressive symptoms and their severity. Scoring ranges from 0 to 63 (0 to 13 indicates no or very few depressive symptoms, 14 to 19 mild depression, 20 to 28 moderate depression, and 29 to 63 severe depression). The BDI-II has good reliability and validity and has shown high internal consistency [25].

#### Secondary outcomes

##### Perceptions of caregiver role

The Carers of Older People in Europe (COPE) [26]. Index is an assessment of carers’ perceptions of their role as a caregiver. A total of 13 questions assess the negative impact (seven items), the positive value of caregiving (four items) and the quality of support (four items). Each item is rated on a four-point scale: never, sometimes, often, and always, that indicates the degree of the caregiver’s personal experience of the aspect of caregiving in question. Higher scores on the negative aspects (scores 7–28) indicate a more negative impact of caregiving, and higher scores on both the positive aspects and quality of support (scores 4–16) indicate a more positive impact and better quality of support in caregiving. The COPE Index was developed in collaboration with several European countries as a brief assessment tool for identifying caregivers who may need supportive interventions. Cronbach α for COPE Index is .87 [27].

##### Anxiety

Generalized Anxiety Disorder 7-item (GAD-7) [28] scale is a self-report questionnaire for assessing generalized anxiety disorder. The GAD-7 comprises seven items measuring the severity of various signs of GAD. The response categories are scored from 0 (not at all) to 3 (nearly every day). A sum score (min 0, max 21) is calculated. Scores of 5–9 points indicate moderate anxiety, 10–14 moderate and > 15 severe anxiety. Higher GAD-7 scores correlate with disability and functional impairment. The 7-item anxiety scale has good reliability and validity [28].

##### Quality of life

WHO Quality of Life-BREF (WHOQOL-BREF) is an abbreviated version of the WHOQOL-100 [29] and produces a quality of life profile based on assessment in four domains: physical health, psychological health, social relationships, and environment. The WHOQOL-BREF contains 26 questions, scored from 1 to 5 (1 = not at all, very poor, 5 = completely, very good, very satisfied). Each domain is scored separately. Separate scores are also given for two of the 26 items: self-perceived overall quality of life (question 1) and health (question 2). Higher domain scores indicate higher quality of life. To render the domain scores comparable with the scores used in the WHOQOL-100, they are each multiplied by 4 [30]. Analyses indicate that the WHOQOL-BREF has good to excellent psychometric properties of reliability and validity [29].

##### Sense of coherence

Sense of coherence will be measured by the 13-item Orientation to Life -Questionnaire which is an abbreviated version of the original 29-item scale measuring different aspects of sense of coherence (SOC) [31, 32]. The scale comprises 13 items from 1 (= rarely or never true) to 7 (= true most of the time). Sum score thus range from 13 to 91. The scale consists of three dimensions: Comprehensibility (items 2, 6, 8, 9, 11; min 5, max 35), Manageability (items 3, 5, 10, 13; min 4, max 28), and Meaningfulness (items 1, 4, 7, 12; min 4, max 28). The scores on the three subscales are then summed into a total score, with higher scores indicating better outcomes. The scale has been found to be reliable, with Cronbach’s α ranging from .75–.91 [32].

##### Psychological flexibility

Psychological flexibility will be assessed using the Acceptance and Action Questionnaire (AAQ-II) [33]. The AAQ-II measures experiential avoidance (EA) and is a shorter version of the AAQ-16 and AAQ-10 –versions [34]. It comprises seven items to be answered on a scale of 1 (never true) to 7 (always true) on the person’s willingness to be in contact with negative private events, acceptance of these events, and whether they can live in accordance with their values. Sum scores range from 7 to 49, with higher score indicating a worse outcome, i.e. more experiential avoidance and less psychological flexibility [33]. The scale indicates satisfactory structure, reliability, and validity [33], with Cronbach’s α ranging from .78–.88.

##### Experiential avoidance in caregiving

The Experiential Avoidance in Caregiving Questionnaire (EACQ) [35] is a scale measuring experiential avoidance in the caregiving context: (1) Active Avoidant Behaviors; (2) Intolerance of Negative Thoughts and Emotions Towards the Relative; and (3) Apprehension Concerning Negative Internal Experiences Related to Caregiving. The questionnaire comprises 15 items each rated from 1 (not at all) to 5 (a lot). Factor 1 contains 6 items that measure caregivers’ behaviors for avoiding negative thoughts related to caregiving (min 5, max 25). Factor 2 contains 4 items with content related to rigid verbal thinking about having negative emotions/thoughts about the care recipient (min 4, max 20), and factor 3 contains 5 items referring to reluctant attitudes towards negative thoughts about the care recipient (min 5, max 25). Sum scores are calculated for the three subscales, with higher scores indicating more avoidance, e.g. worse outcome. The EACQ shows acceptable psychometric properties, with Cronbach’s α for the total scale being .70 [35].

##### Thought suppression

The White Bear Suppression Inventory (WBSI) is a 15-item questionnaire designed to measure thought suppression. Chronic thoughts suppression is related to obsessive thinking and negative affect associated with depression and anxiety [36]. The WBSI is rated on a 5-point scale from Strongly disagree (1) to Strongly agree (5). Total scores range from 15 to 75 with higher scores on the WBSI indicate greater tendencies to suppress thoughts. Studies show that the WBSI is a reliable and valid self-report instrument [37].

##### Personality

The ‘Short Five’ (S5) personality inventory is a 60-item questionnaire constructed for measuring 30 facets of the Five-Factor Model [38]. The personality traits measured are the Big Five: neuroticism, openness, conscientiousness, extroversion, and agreeableness [38]. Each item is rated on a 7-step scale from − 3 to + 3. The inventory comprises 30 question pairs, six pairs, i.e. 12 questions for each trait. Questions are scored in pairs so that the score for the second question is deducted from the score for the first question. Sum scores, ranging from − 36 to 36, are calculated for each trait. S5 provides more detailed information than most of the other scales of similar length, and shows better reliability and convergent validity than even shorter questionnaires [38].

##### Physical performance

The Short Physical Performance Battery (SPPB) combines the results of the gait speed, chair stand and balance tests [20]. It has been used as a predictive tool for possible disability and can aid in the monitoring of function in older people. The scores range from 0 (worst performance) to 12 (best performance). The SPPB has been shown to have predictive validity showing a gradient of risk for mortality, nursing home admission, and disability. Studies show that the SPPB is a reliable and valid instrument [39]. In this sample, physical performance was measured at pre-measurement for all groups, and in addition for the web-based group at four and 10-months.

##### Adherence, user experiences, and usage of technology

The post-measurement includes a user experience questionnaire for the web-based intervention group. The questionnaire developed by the research group assesses usability, acceptance, perceived benefits, and usage of the web intervention. The first five questions are rated on a 10-point scale from 1 to 10: 1 = very unsatisfied, 10 = very satisfied (item 1); 1 = not at all important, 10 = very important (items 2 and 4); 1 = very satisfied, 10 = not at all satisfied (item 3); 1 = has deteriorated, 10 = has improved remarkably (item 5). Item 6 assesses current well-being on a 7-point scale, item 7 time spent weekly in the web program on a 4-pont scale (less than 1 h, between 1 and 2 h, 2–3 h and more than 3 h), and the remaining two questions are open-ended questions. The questionnaire also includes open-ended questions such as *What was it like to participate in this program? What have you learned from this program? Was the program easy to use?* The post-measurement will also include interviews for collecting qualitative data that can provide deeper insights into user experiences and perceived benefits. Objective technology usage data will be extracted from log files generated by the web application.

### Statistical analysis

The sample size for the trial was estimated based on the change in depressive symptoms (the primary outcome) measured by the BDI-II in a previous study [40]. A sample size of 50 elderly family caregivers in each of 1) an Acceptance and commitment therapy –based online psychological intervention group (experimental group), and 2) a standardized institutional caregiver rehabilitation program (active comparison group representing usual care), and 3) an active control group receiving support from a caregiver associations receiving support provided by voluntary caregiver associations was calculated as the minimum required to detect a 5.8 point decrease in BDI-II in 1) the ACT intervention group or 2) the standardized institutional caregiver rehabilitation program vs. no change in 3) the caregiver support group at the 10-month post-measurement to achieve 80% power. The calculations were based on a one-sided General Estimation equation (GEE) model [41] using the pre-measurement, four-month and 10-month post-intervention measurements. We assumed that the post-measurement difference between the 1) intervention or 2) usual care group vs. 3) caregiver support group would be 5.8 points (*SD* = 4.8) lower than at pre-measurement (9.3 points, *SD* = 7.8). Thus, assuming an attrition rate of 20%, a sample size of 123 (41 per group) was expected. Hence the target sample size was 150 participants, with 50 in each group.

Statistical analyses will be conducted using Mplus (version 7) [42] and SPSS IBM version 24. Analyses will be performed on all participants. Baseline differences between the groups will be examined by using t-tests and chi-square -tests. Data will be analyzed using the IBM SPSS statistics version 22.0 or newer and Mplus statistical package 7.1. Analyses will be controlled for possible baseline differences between the groups. Between group differences in the stability of the outcome measures will be examined using multiple-group modeling techniques. Full information maximum likelihood (FIML) estimation on the assumption of data missing at random (MAR) will be used in analyzing incomplete data. When the normality assumption is violated, maximum likelihood with robust standard errors (MLR) will be used. The level of significance will be set at 0.05. Cohen’s *d* will be used to estimate effect sizes, and to reflect the clinical significance of the interventions.

## Discussion

### The aim of the study

This article describes a research protocol for a quasi-experimental trial investigating whether a guided web-based acceptance and commitment therapy (ACT) intervention (Group 1) or a standardized rehabilitation program provided by rehabilitation centers (Group 2) is more effective in reducing depressive symptoms and improving the well-being and quality of life of elderly family caregivers, compared to support provided by voluntary family caregiver associations (Group 3). A further aim is to investigate whether personality, psychological flexibility or other psychological variables predict, or mediate the effects of the web-based ACT intervention. User experiences and the satisfaction of family caregivers with the web-based intervention will also be examined. We expect the effect of the web-based intervention to be equal to the effect of the established comparator group (Group 2), and show more positive effects on depressive symptoms and well-being than the support provided by family caregiver associations (Group 3).

Family caregivers who provide complex chronic care play a crucial role in the long-term care of older adults worldwide. Several studies have shown, however, that while caring for a person with a long-term condition or disability may be rewarding, it may also have serious negative effects on the caregivers’ physical and mental health [43, 44]. Depression, stress and other types of psychological distress are major health concerns among family caregivers [5]. It is, therefore, of great importance to help to decrease the negative impact of caregiving on family caregivers. Low intensity interventions combined with technology enable 24/7 committed and stressed caregivers to participate when they have both the time and need to do so. However, it is important to evaluate the acceptability of technology-based interventions and their effects on psychological and physiological variables.

This study will provide knowledge on the effectiveness and acceptability of a web-based intervention aimed at decreasing depressive symptoms and increasing well-being and quality of life among caregivers aged 60 or more. We compare a web-based intervention with a standardized institutional rehabilitation and support provided by family caregiver associations. While the current institutional rehabilitation program has successfully met the caregivers’ physical needs, results show that it has not improved caregivers’ mental health [45]. If the web-based CareACT intervention improves family caregivers’ mental health and if their physical functioning is also found to be on an acceptable level for independent living, the web-based intervention should be made available to all family caregivers. If support received from family caregiver associations does not show changes comparable with those of the web- and institutional programs in reducing depressive symptoms, it may nevertheless offer a potential environment for implementation of the CareACT program which has proven effectiveness. In sum, this study will increase our understanding of the effectiveness and suitability of acceptance, mindfulness and value-based brief interventions for elderly family caregivers suffering from depressive symptoms and psychological distress.

### Strengths

This study has several strengths. First, it includes a relatively large number of participants from different regions of the country. Moreover, it utilizes many psychological measurements in addition to assessments of balance and lower extremity muscles. Physical function is an important clinical measure, which assesses the ability of family caregivers to cope in their daily life at home. This study will also provide novel data on the psychological change processes associated with guided web-based interventions and their outcome. This research project will show whether, and if so, which ACT methods produce meaningful change and whether the processes of change indicated by measures of psychological flexibility differ across different approaches.

### Limitations

This study has its limitations. Firstly, we are unable to conduct the study as a randomized controlled trial. Instead, the study is a clinical R & D project and designed to investigate technology that could provide family caregivers with new and easy accessible means of support in their caregiving work and improve their well-being. Standardized rehabilitation is institutionalized and organized by the Finnish Social Insurance Institution. Access to rehabilitation follows a strict standardized procedure that does not allow randomization. Second, the recruitment protocol was different in each study group. The experiential web-based sample was recruited via a newspaper advertisement. Responders to a newspaper advertisement may be selected and more motivated for lifestyle change than family caregivers in general, which could be a cause of selection bias in our analyses. A further limitation is that the caregiver association support group (Group 3) did not undergo a screening procedure similar to that applied in selecting the other groups at the beginning of the study. However, the support group was used as a control group, which helps to reflect the real state of psychological health and well-being of family caregivers in different regions.

This study will provide knowledge on effects of a novel web-based acceptance and commitment intervention and of a standardized institutional rehabilitation program representing usual care for reducing depressive symptoms and improving the psychological well-being of elderly family caregivers and to a control group receiving support offered by voluntary family caregiver associations. If successful, the study will yield information on the persons for whom these interventions would be most beneficial, and what mechanisms mediate the intervention effects. The results expand the knowledge base of clinicians and adduce evidence on effective strategies for improving the mental health, physical function and quality of life of elderly family caregivers. The results will also assist decision- and policy-makers to develop rehabilitation strategies and guidelines, and so provide family caregivers in need of increased psychosocial support with provenly effective services.

## Abbreviations

AAQ-II: Acceptance and Action Questionnaire
ACT: Acceptance and Commitment Therapy
BDI-II: Beck Depression Inventory
CBT: Cognitive Behavioral Therapy
COPE: Carers of Older People in Europe
DEPS: Depression Scale
EACQ: Experiental Avoidance in Caregiving Questionnaire
FIML: Full information maximum likelihood
GAD-7: Generalized Anxiety Disorder
GEE: General Estimation equation model
MAR: (data) missing at random
MLR: maximum likelihood with robust standard errors
R&D: Research & Development
S5: The Short Five
SII: The Social Insurance Institution of Finland
SOC-13: Sense of Coherence
SPPB: Short Physical Performance Battery
WBSI: White Bear Suppression Inventory
WHOQOL-BREF: WHO Quality of Life - BREF
